# Taurine, a Component of the Tear Film, Exacerbates the Pathogenic Mechanisms of *Acanthamoeba castellanii* in the Ex Vivo Amoebic Keratitis Model

**DOI:** 10.3390/pathogens12081049

**Published:** 2023-08-16

**Authors:** Lizbeth Salazar-Villatoro, Bibiana Chávez-Munguía, Celia Esther Guevara-Estrada, Anel Lagunes-Guillén, Dolores Hernández-Martínez, Ismael Castelan-Ramírez, Maritza Omaña-Molina

**Affiliations:** 1 Departamento de Infectómica y Patogénesis Molecular, Centro de Investigación y de Estudios Avanzados del IPN, Ciudad de Mexico 07360, Mexico; lsalazar@cinvestav.mx (L.S.-V.); bchavez@cinvestav.mx (B.C.-M.); lagunes402@gmail.com (A.L.-G.); 2 Laboratorio de Amibas Anfizóicas, Facultad de Estudios Superiores Iztacala, Universidad Nacional Autónoma de México, Tlalnepantla 54090, Mexico; celiaestherguevara@gmail.com (C.E.G.-E.); mdhm_1965@iztacala.unam.mx (D.H.-M.); ismaelc.40@gmail.com (I.C.-R.)

**Keywords:** *A. castellanii*, taurine in tear, pathogenic mechanisms

## Abstract

*Acanthamoeba* spp. is the etiological agent of amoebic keratitis. In this study, the effect of taurine in physiological concentrations in tears (195 μM) on trophozoites of *Acanthamoeba castellanii* through the ex vivo amoebic keratitis model was evaluated. Trophozoites were coincubated with the Syrian golden hamster cornea (*Mesocricetus auratus*) for 3 and 6 h. Group 1: Control (−). Corneas coincubated with amoebic culture medium and taurine. Group 2: Control (+). Corneas coincubated with trophozoites without taurine. Group 3: Corneas coincubated with taurine 15 min before adding trophozoites. Group 4: Trophozoites coincubated 15 min with taurine before placing them on the cornea. Group 5: Corneas coincubated for 15 min with trophozoites; subsequently, taurine was added. Results are similar for both times, as evaluated by scanning electron microscopy. As expected, in the corneas of Group 1, no alterations were observed in the corneal epithelium. In the corneas of Group 2, few adhered trophozoites were observed on the corneal surface initiating migrations through cell junctions as previously described; however, in corneas of Groups 3, 4 and 5, abundant trophozoites were observed, penetrating through different corneal cell areas, emitting food cups and destabilizing corneal surface in areas far from cell junctions. Significant differences were confirmed in trophozoites adherence coincubated with taurine (*p* < 0.05). Taurine does not prevent the adhesion and invasion of the amoebae, nor does it favor its detachment once these have adhered to the cornea, suggesting that taurine in the physiological concentrations found in tears stimulates pathogenic mechanisms of *A. castellanii*.

## 1. Introduction

*Acanthamoeba* spp. are ubiquitous in nature. Several species of the genus are considered amphizoic amoebae because they can exist in free-living form and eventually cause infections in humans, being recognized as etiological agents of granulomatous amebic encephalitis, skin lesions and corneal infection. For this reason, these protozoa have gained medical importance in recent years. In particular, *Acanthamoeba* keratitis (AK) is a progressive sight-threatening corneal infection that can occur both in immunocompromised and healthy individuals, being prevalent among contact lens wearers [1,2]. Early diagnosis and adequate therapy are essential to ensure a good prognosis [3,4,5].

It has been established that the pathogenesis of AK infection is a multistep process in which contact-dependent mechanisms play a relevant role during the invasive processes of these amoebae [6]. In vitro and in vivo studies have been undertaken to describe the pathogenic mechanisms of different species of the *Acanthamoeba* genus (*A. castellanii*, *A. culbertsoni*, *A. royreba*, *A. griffini* and *A. polyphaga*), both in hamster and human corneas, as well as in MDCK cells [7]; it has been established that a single trophozoite or small groups of them firmly adhere to intact as well as damaged corneas, migrating to the paracellular space of superficial corneal cells. After a few hours post interaction, amoebae penetrate the deeper layers of corneal tissue, inducing the disruption of normal corneal epithelium architecture and emitting food cup structures to ingest detached epithelial cells [8]. In addition, amoebae continue migrating into the deeper layers of the corneal epithelium, and only those that show greater virulence are able to invade the corneal stroma 12 h after being placed on the corneal surface [9].

Despite the advances in the description of *Acanthamoeba* spp. invasion mechanisms, studies performed in vitro and ex vivo may not emulate the events that take place in vivo in which the ocular surface is regularly exposed to the external environment and potential pathogens.

The first line of defense amongst the external environment and the ocular surface is the tear film that covers the eye, which contains a variety of humoral defense components such as lactoferrin, lysozyme and lactoperoxidase [10]. As the major immunoglobulin isotype in normal tears, lgA constitutes a frontline defense against microorganisms [11,12]. Several studies have suggested that the mucosal immune system provides protection against *Acanthamoeba* keratitis [13,14]. In addition to this, it has been reported that some tear components prevent several pathogens from invading the ocular surface; Tomita et al. [15] demonstrated in vitro lactoferrin amoebicidal capacity only in the trophic phase of *Acanthamoeba*; nevertheless, it has been reported that the activity of lysozyme and lactoferrin does not prevent the adhesion of *Acanthamoeba* to the corneal epithelium [16].

In 2011, Nakatsukasa et al. [17] reported 23 amino acids in human tears, associating their decrease with ocular surface diseases, where the most abundant amino acid is taurine (195.1 +/− 26.9 µM). The role of taurine (2-aminoethane sulfonic acid) in humans has been studied, highlighting its participation in cytoprotection mechanisms, acting as an epithelial barrier [18,19]; it also acts as a neurotransmitter, osmoregulator and antioxidant in visual and neuronal development, as well as being involved in metabolic and inflammatory processes [20].

In the eye environment, taurine has been suggested for dry eye treatment [21]. In combination with sodium hyaluronate, it has been evidenced that taurine protects corneal epithelium cells, maintaining osmotic pressure and tear volume [20]. Recently, the role of taurine as a chemoattractant has been studied; Talamás-Lara et al. [22] evaluated in vitro neuroactive substances including taurine as an inducer of the migration of *A. castellanii* trophozoites. Until now, it has been unknown if taurine is able to prevent adhesion and invasion of *Acanthamoeba* trophozoites to corneal tissue. In this study, we evaluate the effect of taurine at physiological concentrations reported in the tear film on pathogenic mechanisms of *Acanthamoeba castellanii* trophozoites in the Syrian golden hamster (*Mesocricetus auratus*) ex vivo model of amoebic keratitis.

## 2. Materials and Methods

### 2.1. Amoebae Culture

This study was carried out with *A. castellanii* trophozoites isolated from an amoebic keratitis case diagnosed in the Asociación para evitar la ceguera en México “Luis Sánchez Bulnes Hospital”, Mexico City, grown in the axenic culture medium Bacto Casitone at 2% (pancreatic digest of casein; Becton Dickinson, and company Sparks, MD, USA), supplemented with 10% fetal bovine serum (Biowest No cat. S1650, Mexico City, México) and 1% (*w*/*v*) antibiotics (10,000 U/mL penicillin and 10 mg/mL streptomycin). Assays were performed with trophozoites harvested at the end of the logarithmic growth phase (72 h).

The amoeba under study was identified as *A. castellanii* according to the morphological criteria found in Page, 1988 [23]. Additionally, it was identified as belonging to genotype T4 according to molecular identification carried out by sequencing the diagnostic fragment 3 (DF3) of the 18S ribosomal DNA gene of *Acanthamoeba* T4 [24].

### 2.2. Reactivation of A. castellanii Virulence

Intranasal inoculation in mice was performed to reactivate virulence and confirm the invasiveness capacity of amoebae [25].

Mice were fed ad libitum and monitored daily to observe some signs of infection. After 21 days, the surviving mice were sacrificed. The brain, liver, lungs and kidneys were cultured in agar plates with nonnutritive enriched medium (NNE) to recover the amoebae. The recovered *A. castellanii* trophozoites were axenized similarly to when they were isolated from the clinical case [26,27] and used in the different assays proposed. Experimental animals used for amoebae virulence reactivation were manipulated in accordance with approved standard project number 174 for the reactivation of amphizoic amoebae virulence, supported by the Official Mexican Standard NOM-062-ZOO-1999, based on the Guide for the Care and Use of Laboratory Animals, published in the Official Journal of the Federation (Mexico) 2001.

### 2.3. Qualitative Evaluation of the Interaction of A. castellanii with Taurine in the Ex Vivo Model of Amoebic Keratitis

#### 2.3.1. Surgical Extraction of Hamster Corneas

Adult male Syrian golden hamsters (*Mesocricetus auratus*) weighing 120 to 130 g were used to induce *A. castellanii* cornea interactions. After anesthesia with sodium pentobarbital (5 mg/100 g of body weight), both corneas were removed leaving a peripheral rim of scleral tissue [6,9].

#### 2.3.2. Ex Vivo Interaction of *A. castellanii* Trophozoites with Hamster Cornea

Taurine (2-aminoethane sulfonic acid of Sigma-Aldrich-T8691) was used in this work since its similarity by capillary electrophoresis with taurine present in human tears has been reported [28]. The taurine used in each experimental group was dissolved in Bacto Casitone culture medium and adjusted to a final concentration of 195 µM, as according to Nakatsukasa et al., 2011 [17]. Isolated corneas were washed with PBS to clean the corneal surface, then placed in 96-well styrene plates (Corning Incorporated, Corning, NY, USA). *A. castellanii* trophozoites were adjusted to 2.5 × 10^5^/150 μL culture medium and were incubated according to the following experimental strategy:

- Group 1: corneas coincubated with amoebic culture medium and taurine dissolved in the medium. Control (−).
- Group 2: corneas coincubated with *A. castellanii* trophozoites. Control (+).
- Group 3: corneas coincubated with taurine interacting 15 min before adding trophozoites of *A. castellanii*.
- Group 4: *A. castellanii* trophozoites interacting for 15 min with taurine before placing them on the cornea, in order to determine if taurine is capable of preventing the adhesion of trophozoites to the corneal epithelium.
- Group 5: corneas interacting with *A. castellanii* for 15 min before subsequently adding taurine, to determine if taurine has any effect on trophozoites once they have adhered to the corneal epithelium.

All assays were incubated at 36.5 °C since the human cornea temperature ranges around 37 °C with a variance between 0.5 and 1 °C. The interaction was carried out for 3 and 6 h [29]. Samples were then fixed and processed for scanning electron microscopy. All assays were performed in triplicate.

#### 2.3.3. Scanning Electron Microscopy (SEM)

The morphological alterations of the cornea induced during the invasion of *A. castellanii* trophozoites in the proposed experimental groups in the ex vivo model of amoebic keratitis were analyzed by SEM. After incubation, the samples were fixed with 2.5% glutaraldehyde in 0.1 M cacodylate buffer with pH 7.2, washed with distilled water and dehydrated with increasing concentrations of ethanol. Samples were critical-point dried with liquid CO_2_ using a Samdri 780 apparatus (Tousimis Research Corp.) and coated with a thin layer (30 nm) of gold in an ion-sputtering device (Jeol-JFC I 100). Hamster corneas were examined with a JEOL-JSM 7100 F scanning electron microscope (JEOL Ltd., Tokyo, Japan).

### 2.4. Quantitative Evaluation of the Interaction of A. castellanii with Taurine in the Ex Vivo Model of Amoebic Keratitis

With the purpose of determining possible *Acanthamoeba* adhesion differences induced by taurine, the number of amoebae adhered to the corneal surface was quantified in each of the experimental groups. Five distant areas of panoramic views obtained from the scanning electron microscope (300×) were counted, trying to cover the corneal surface as widely as possible.

Statistical analysis was performed obtaining the arithmetic means of trophozoites adhered to the corneal surface from each experimental group. The statistical significance of the effect of taurine on the adhesion of *A. castellanii* trophozoites was analyzed through Student’s *t*-test using GraphPad Prism 6 software (San Diego, CA, USA). Differences with a *p* value < 0.05 were considered significant.

## 3. Results

### 3.1. Reactivation of A. castellanii Virulence

The strain under study was inoculated in mice to reactivate its virulence, recovering from the brain and lung. *A. castellanii* caused the death of 60% of the experimental animals during the first 11 days post inoculation. The studied strain was shown to be invasive in the in vivo GAE mice model, and in the ex vivo hamster AK model it caused evident damage to the corneal epithelium. All subsequent assays were carried out with these recovered and axenized amoebae.

### 3.2. Qualitative Evaluation of the Interaction of A. castellanii with Taurine in the Ex Vivo Model of Amoebic Keratitis

Interaction of *A. castellanii* with taurine in the ex vivo model of amoebic keratitis was analyzed at proposed times of 3 and 6 h in the different experimental groups through scanning electron microscopy. It is important to mention that the results observed in both times were very similar; thereby, the results are presented indistinctly.

**Figure 1:**
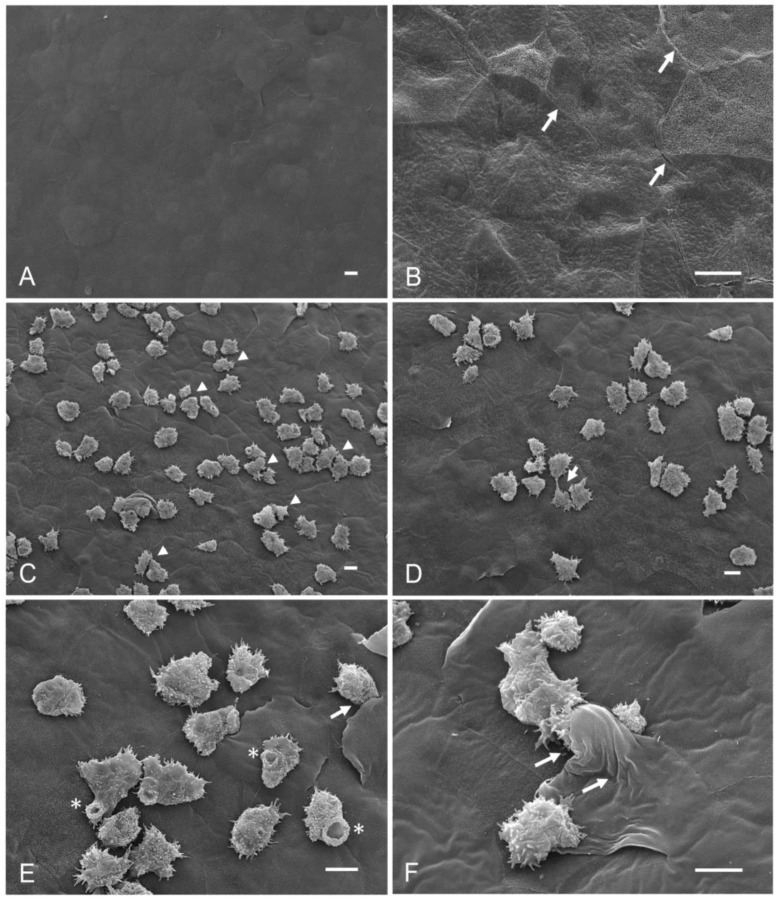
Scanning electron microscopy. Qualitative evaluation of the interaction of *A. castellanii* in the ex vivo model of amoebic keratitis. (**A**,**B**) Group 1, control corneas interacting for 3 and 6 h at 36.5 °C with amoebic culture medium to which 195 µM of taurine was added; an intact corneal epithelium is observed. (**B**) At higher magnification, well-defined cell junctions are visualized (arrows). (**C**–**F**). Group 2, corneas coincubated with 2.5 × 10^5^ trophozoites of *A. castellanii* without taurine. (**C**) A considerable number of trophozoites adhered to the corneal surface are observed (arrowheads). (**D**) Trophozoites mainly attached to the cell junctions of the cornea; amoebae are observed in the process of cell division (arrow). (**E**,**F**) It is possible to see trophozoites emitting food cups (asterisks) and penetrating the cell junctions; the folding and lifting of the corneal cells are noted (arrow). Bar = 10 μm.

Group 1 (control −) corresponded to corneas interacting with culture medium and taurine; both the structure and continuity of the corneal surface were preserved, and the borders between one cell and another well defined, without de-epithelialized areas or abrasion (Figure 1A,B). In Group 2 (control +), control corneas interacting with *A. castellanii*, trophozoites were observed adhering to the corneal epithelium with their classic amoeboid shape and their characteristic acanthopodia (Figure 1C); situated mainly on the cornea cells’ junction zones, it was also possible to see amoebae in the process of cell division (Figure 1D). Some trophozoites were observed emitting food cups and a small amount of trophozoites were observed penetrating between the cell junctions; likewise, the folding and detachment of the surface cells were observed in areas of amoebic invasion (Figure 1E,F).

**Figure 2:**
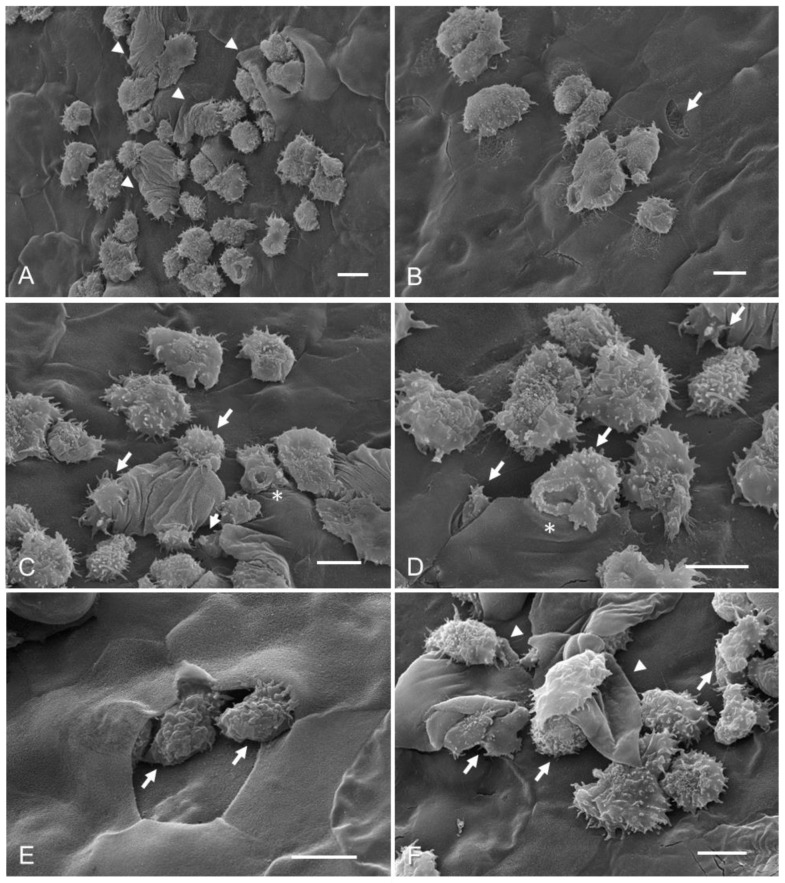
Hamster corneas coincubation with taurine, and subsequent interaction with trophozoites of *A. castellanii* (Group 3). (**A**) A considerable number of trophozoites are observed adhering and penetrating the corneal epithelium, causing evident damage to the corneal surface (arrowhead). (**B**) An abrasion zone (arrows) is observed adjacent to a group of trophozoites adhered to the corneal epithelium. (**C**,**D**) A large number of amoebae penetrating below the corneal cells, which probably causes the detachment of the superficial cells of the corneal epithelium (arrows), suggesting the occurrence of the process of phagocytosis coupled with the presence of food cups in some trophozoites (asterisks). (**E**,**F**) It is possible to observe areas devoid of cells and the presence of amoebae below the first cell layer (arrows). The detachment of cells from the corneal epithelium is evident at 3 and 6 h of interaction as is a visible alteration in the corneal surface (arrowheads). Bar = 10 μm.

**Chart 1:**
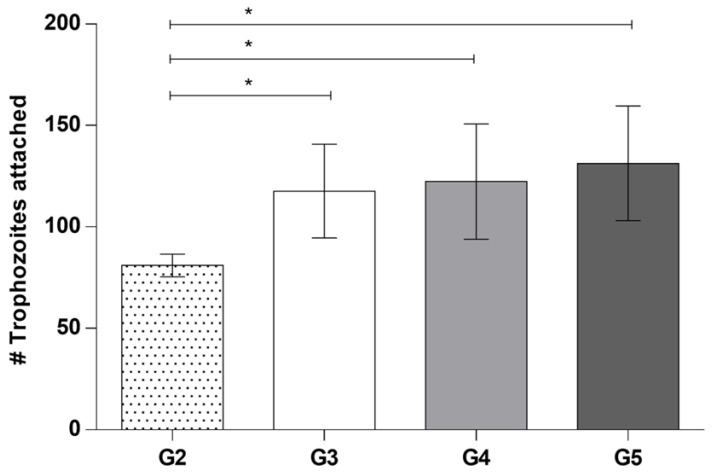
Quantitative evaluation of the interaction of *A. castellanii* with taurine in the ex vivo model of amoebic keratitis. Student’s *t*-test was performed using GraphPad Prism 6 software and indicated statistically significant differences in the number (#) of trophozoites that adhered to control Group 2 compared to experimental Groups 3, 4 and 5 coincubated with taurine * *p* < 0.05.

Concerning Group 3, in which corneas were coincubated with taurine before adding trophozoites of *A. castellanii*, the purpose was to determine if taurine protects corneal epithelium cells against amoebic invasion; however, an increase in the number of adhered amoebae was observed on the corneal surface compared to Group 2 (control +) (Chart 1), as well as damage to the corneal surface (Figure 2A). Corneal injury areas were observed in adjacent cell junction zones, possibly caused by trophozoites of *A. castellanii* (Figure 2B). Corneal surface protrusions associated with the invasion of numerous trophozoites were observed, as well as areas of detached cells. It was possible to visualize the constant presence of food cups in a large number of trophozoites, suggesting a persistent phagocytosis process (Figure 2C–E). Several trophozoites invaded the epithelial cell layer, protruding or destroying the corneal surface in which amoebae were observed beneath the superficial cells, modifying the corneal architecture (Figure 2E,F).

**Figure 3:**
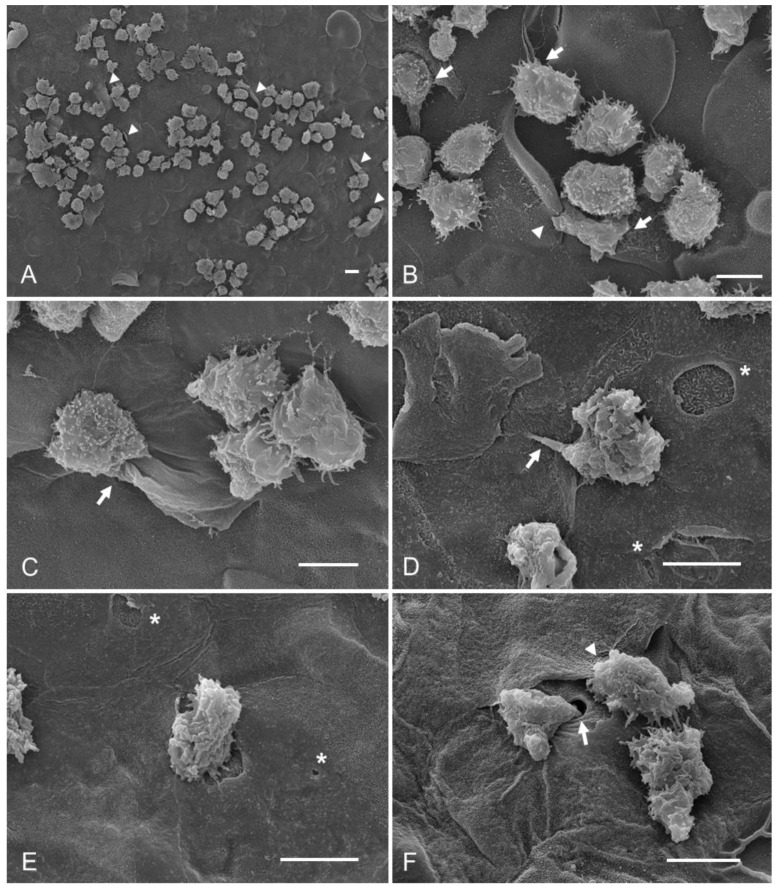
*A. castellanii* trophozoites coincubated with taurine, and their subsequent interaction with hamster corneas (Group 4). (**A**) An abundant number of trophozoites adhered to the corneal surface are observed; areas with damage are visible (arrowheads). (**B**) A group of amoebae is found penetrating between the cell junctions of the corneal surface (arrows) by mechanical and/or enzymatic action; it is possible to appreciate the formation of a food cup of an amoeba surrounding a portion of a cell epithelial (arrowhead). (**C**–**E**) the phagocytosis process is evident; some trophozoites were observed emitting food cups; one of them was prominent, the amoeba engulfing a cell on the surface of the cornea just at the cell junction, and another amoeba emitted very thin food cups, ingesting a portion of the corneal cell away from the cell junction (arrows); there were also visible abrasion areas far from cell junctions (asterisks). (**F**) It was possible to observe a trophozoite invading part of the amoebic body to penetrate deeper layers of the cornea (arrow). Similarly, an amoeba in the process of phagocytosis of a corneal epithelial cell can be seen (arrowhead). Bar = 10 μm.

To evaluate the effect of taurine on trophozoites and determine its possible role in preventing their adherence to the corneal epithelium, in Group 4, *A. castellanii* trophozoites were coincubated with taurine before placing them on the cornea. Abundant trophozoites adhered to the corneal surface compared to the control group were observed (Chart 1). Areas of damage to the corneal epithelium were evident, as well (Figure 3A). Some amoebae were observed to be located on the cell junctions, some penetrating through these; the phagocytosis processes of recently detached epithelial cells were also evident (Figure 3B). It is important to highlight that some amoebae were observed causing damage in adjacent areas, far from cell junctions, i.e., areas in which amoebae usually had been observed to initiate invasion, which does not correspond to the pathogenicity mechanisms reported previously. It is important to highlight that food cups of different sizes and forms were frequently observed; since trophozoites were observed emitting large food cups and fine phagocytic projections (Figure 3C), the corneal surface showed abrasion and a cytopathic effect without previous detachment of the corneal epithelial cells; it was common to observe areas of damage far from the cell junctions (Figure 3C,D). It is also noteworthy that the amoebae were visualized introducing part of the amebic body to and invading the deeper layers of the epithelial cornea. Moreover, abrasion areas were observed through which the trophozoites penetrate without implying a previous detachment of the epithelial cells (Figure 3E,F).

**Figure 4:**
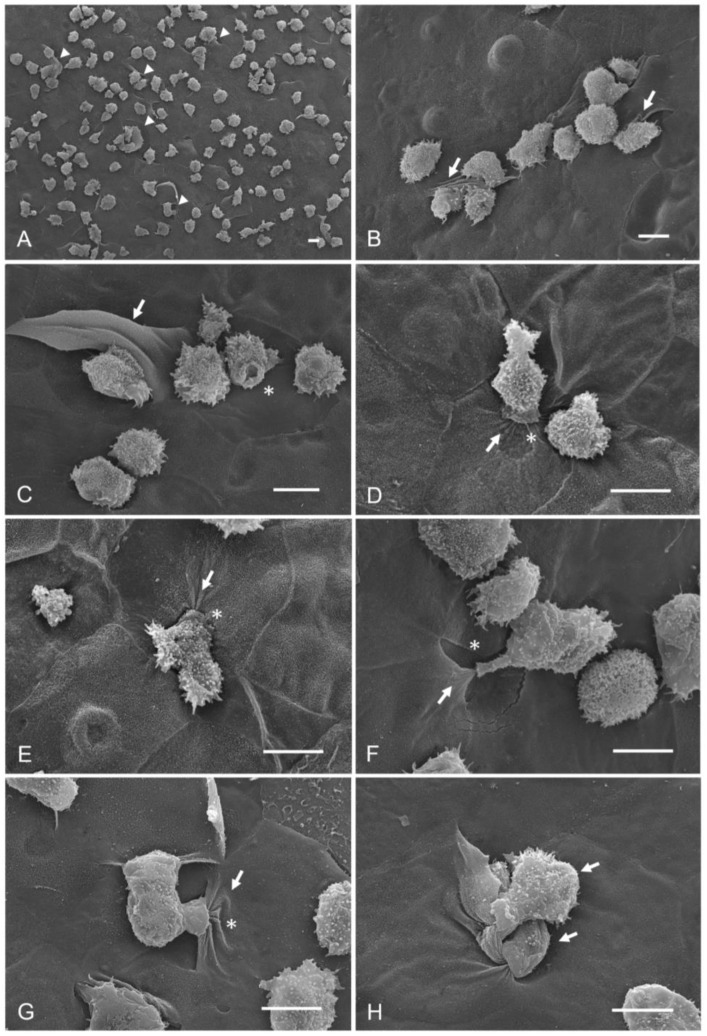
Interaction of trophozoites of *A. castellanii* with corneas and subsequent taurine addition (Group 5). (**A**) A large number of adherent *A. castellanii* trophozoites, as well as areas of damage, are visible on the corneal surface (arrowheads). (**B**,**C**) Amoebae are observed adhering to the corneal surface, emitting their characteristic acanthopodia. The trophozoites of *A. castellanii* alone or in groups initiate the disorganization of the superficial cells of the cornea, which are observed to be folded (arrows) as a consequence of the mechanical action exerted by the amoebae; a food cup is observed (asterisk). (**D**–**G**) Several trophozoites are in the process of phagocytosis of corneal epithelial cells in areas far from the cell junctions which have not yet been detached (arrow). It is possible to observe the development of various food cups (asterisks) and a great avidity of the trophozoites for cells of the corneal epithelium. (**H**) It was possible to observe the phagocytosis of an epithelial cell by two trophozoites (arrows) at the same time. Bar = 10 μm.

Finally, in Group 5, the effect of taurine on the trophozoites previously adhered to the corneal surface was determined. Abundant trophozoites attached to the corneal epithelium were observed, as well as areas of damage in which trophozoites have begun to invade through the spaces between one cell and another (Figure 4A). In the same way, small groups of amoebae were observed initiating the invasion process on the corneal surface (Figure 4B,C). It is important to note that the corneal epithelial cell phagocytosis process was observed in areas far from the cell junctions, without the amoeba previously invading the corneal surface; specifically, amoebae induced the cytopathic effect without previously detaching the corneal surface cells, contrary to what has usually been reported during the invasion of these amoebae. An increase in the avidity of *A. castellanii* trophozoites to phagocyte corneal epithelium cells even without being detached was evidenced (Figure 4D–H).

### 3.3. Quantitative Evaluation of the Interaction of A. castellanii with Taurine in the Ex Vivo Model of Amoebic Keratitis

The statistical significance of the number of adhered trophozoites to the hamster cornea and the possible effect on adhesion of taurine were determined. The results of the quantitative analysis are shown in Chart 1, where the highest numbers of adhered amoebae were observed in the experimental Groups 3, 4 and 5. Student’s *t*-test using GraphPad Prism 6 software indicated significant differences in the adherence of trophozoites from control Group 2 (control +) compared to groups 3, 4, and 5 coincubated with taurine * *p* < 0.05, which strongly suggests the effect of taurine on the adherence of *A. castellanii*.

## 4. Discussion

In our study, we carried out the assays with *A. castellanii* isolated from a clinical case, which corresponds to one of the most frequently isolated species in cases of AK [30,31].

The early associated pathogenicity mechanisms implicated with AK have been reported to consist of trophozoite adherence through the interaction of adhesins, mannose-binding proteins (MBP) and laminin-binding proteins (AhLBP), which interact with glycoproteins and corneal surface glycolipids [32,33,34]. Single trophozoite or small groups of them adhere to the corneal surface, migrating to the paracellular space of superficial corneal cells. Afterward, trophozoites—possibly through mechanical effects and/or enzymatic processes—move into the intracellular space forming bumps, causing cell detachment [6]. Early in the post-interaction period, amoebae penetrate the deeper layers of corneal tissue, inducing the disruption of the normal architecture of the corneal epithelium. Amoebae continue migrating into deeper layers of the desquamating epithelia, forming structures to ingest detached epithelial cells [7,8,35,36]. This process has been corroborated in several species of the *Acanthamoeba* genus, as well as different experimental models and target tissues, with very similar results in all of them [6,29].

In addition, it is important to highlight that phagocytosis plays an important role in the pathogenicity of *Acanthamoeba*. Allen and Dawidowicz [37] reported that *Acanthamoeba* phagocytosis is mediated by receptors that identify mannose in the surface target cells. Likewise, Schuster and Levandowsky [38] reported the presence of chemical signal receptors sensitive to diverse peptides and bacterial products in the plasma membrane of these amoebae, which induce a locomotive response.

*Acanthamoeba* phagocytosis induces damage to the target tissue, which consists of the emission of different phagocytic structures (food cups) in contact with the target tissue [8,39], through which amoebae destroy the architecture of the corneal epithelium. The formation of phagocytic mouths (amoebostomes) or trogocytosis have also been implicated in the pathogenicity of *E. histolytica* [40] and *N. fowleri* [30,41], respectively. In this study, an increase in phagocytic processes was evidenced during the interaction of the amoebae under study with taurine.

Despite the advances in the description of the pathogenic mechanisms of *Acanthamoeba* spp. through in vitro and ex vivo studies, these do not emulate events that take place in vivo in which the ocular surface is regularly exposed to the external environment and potential pathogens. Moreover, faced with corneal invasion by *Acanthamoeba* or any other pathogen that attempts to colonize the ocular surface, there are host defense mechanisms in which the first line of defense between the external environment and the ocular surface is the tear film [10]. The lgA is the major immunoglobulin isotype in normal tears constituting a frontline defense against microorganisms [11,12]. Several studies have suggested that the mucosal immune system provides protection against *Acanthamoeba* keratitis [13,14].

Furthermore, the tear film is constituted by a variety of elements including lipids, proteins, mucins, electrolytes [42] and free amino acids [21]. Nakatsukasa et al. [17] reported the presence of 23 amino acids in human tears, where the most abundant amino acid is taurine, and associated their decrease with ocular surface diseases. The effects and properties of taurine in humans have been studied, and are among the most frequently mentioned cytoprotection mechanisms. Taurine systemically acts as a neurotransmitter, osmoregulator and an antioxidant in visual and neuronal development, as well as in metabolic and inflammatory processes [20].

Fürnkranz et al. [43] carried out studies with N-Chlorotaurine, derived from taurine, demonstrating its amoebicidal effectiveness. In this context, Teuchner et al. [44] determined that N-Chlorotaurine (belonging to the chloramine class) inactivates *Acanthamoeba* in the ex vivo porcine model, producing an amoebicidal effect; although the chemical form of taurine differs from the one used in this study, it shows similarity with taurine present in human tears [28].

In this work, it was determined that *A. castellanii* pathogenicity mechanisms were exacerbated in those groups incubated with taurine. Morphological analysis of the interactions was determined by scanning electron microscopy. Two interaction times of 3 and 6 h were taken into account with three experimental groups with very similar results, which indicates that the invasion process does not vary in the time period evaluated.

Under control conditions—i.e., in the absence of taurine—*A. castellanii* trophozoites were observed adhering to the corneal surface, migrated and were observed located mainly between the cell junctions of the epithelium, specifically in the intercellular spaces. Some amoebae penetrated under the epithelial cell layers, forming protuberances. These findings are in concordance with previous studies showing early events during the invasion of these protozoa to experimental animal corneal surfaces as well as the human cornea [7,29].

As expected, in the control group (−), taurine *per se* did not induce morphological changes on the corneal surface, since its structure and continuity were preserved without de-epithelialized areas or abrasion. Conversely, while interacting with taurine, *A. castellanii* trophozoites exhibited greater adherence. According to the statistical analysis, amoebae interacted with taurine in all groups evaluated in this study, and showed a higher number of trophozoites attached to hamster corneas compared to the control group; therefore, we can infer that taurine exacerbates adherence, an essential mechanism of pathogenicity of *Acanthamoeba* in AK.

In all experimental groups, it was possible to observe trophozoites in small groups adhered in areas far from cell junctions as well as invading the first layer of epithelial cells by SEM. In addition, amoebae were observed below the superficial cells; it should be noted that abrasion zones were visualized through which the trophozoites penetrated without prior detachment of the corneal epithelial cells. The damage to the corneal tissue was evident, and the phagocytosis process was a very frequent event; as is known, phagocytic process has been reported to play an important role in the pathogenicity of *Acanthamoeba* [30,45,46]. In this study, a large number of amoebae were visualized engulfing entire cells without previously detaching them from the corneal epithelium, causing evident damage to the corneal architecture, highlighted by the emission of a large number of food cups of different sizes. Omaña-Molina et al. [6] reported the process of phagocytosis of portions of epithelium cells which were previously detached from the corneal tissue. These findings indicate unusual behavior in these protozoa, most likely due to incubation with taurine, which strongly suggests that the invasion process and damage to the corneal epithelium were exacerbated in the presence of this amino acid.

It is remarkable that, when analyzing the emission of food cups during the interaction of *Acanthamoeba* trophozoites in different target tissues, it has been reported that—in contact with the cornea of either the Syrian golden hamster (*Mesocricetus auratus*) [9], or with the human cornea [7]—trophozoites of different species of the genus *Acanthamoeba* emit large food cups [6]; on the contrary, when these protozoa come into contact with canine kidney cells (MDCK), they emit both large food cups as well as fine projections [36,47]. These fine projections have also been observed during the interaction of *A. castellanii* trophozoites with Schwann cells [48]. This suggests that, depending on the target tissue, possibly caused by the stimulation of surface molecules, the formation of different types of food cups is induced. In this study, it was possible to detect two types of food cups when the trophozoites were stimulated by taurine, large food cups and fine elongated projections with smaller diameters, which reinforces the idea that trophozoites are stimulated by taurine to phagocytize more avidly, emitting different types of food cups.

Moreover, the role of taurine as a chemoattractant has been studied. Talamás-Lara et al. [22] report through in vitro studies that taurine acts as a neuroactive substance that induces the migration of *A. castellanii* trophozoites and also observed phagocytic processes and large adhesion lamellae, suggesting that this amino acid could exacerbate the pathogenicity mechanisms of these amoebae. In addition, Behar et al. [49] suggest that Gamma-aminobutyric acid (GABA) and/or taurine act as chemoattractants of neurons during rat cortical histogenesis, reporting that micromolar concentrations of taurine stimulate migration and act as a chemoattractant for cells in immature cortical regions. Nishiyama et al. [50] reported that *V. cholerae* can be stimulated by taurine, which is the largest component of bile. This suggests that the pathogen senses host factors that allow them to migrate to favorable environments, where the attraction to taurine could play a role in pathogenicity, as well as the chemotaxis of other amino acids in the *Helicobacter pylori* colonization [51]. Baig et al. [52], through bioinformatic tools, reported an effect induced by acetylcholine on *A. castellanii* growth and cell division, and postulated the existence of a muscarinic receptor (hypothetical protein L8HIA6) homologous to that in humans (mAChR1). All this allows us to consider a possible effect of taurine on the trophozoites of *A. castellanii*.

## 5. Conclusions

In conclusion, our observations suggest that taurine does not inhibit or prevent the adherence of trophozoites when pre-incubated, nor does it favor the detachment of those previously attached trophozoites. Contrarily, the disorganization in the corneal architecture of the taurine-interacted corneas was a clear indication of frank damage to the cornea; the qualitative analysis at low magnifications observed through the scanning microscope gave us evidence of a greater number of zones of frank damage in the corneas treated with taurine compared to those that interacted only with trophozoites; it was possible to frequently observe the process of phagocytosis and even trogocytosis, as well as cell detachment, not specifically in cell junctions but also in remote areas. Our research group has previously performed ultrastructural analysis of the hamster cornea where it was possible to demonstrate the migration of trophozoites and especially the damage to the corneal epithelium, with which we can say that this is unusual behavior for *Acanthamoeba* in the presence of taurine, which suggests that taurine in physiological concentrations and without the presence of other components of the tear favors the adhesion and pathogenicity mechanisms of amoebae on the corneal surface, contrary to the idea of the cytoprotective role of taurine. Nevertheless, it is important to analyze the role that other tear components could play, which could be related to protection against AK.

## Data Availability

No new data were created or analyzed in this study. Data sharing is not applicable to this article.
